# Magnetic Resonance Neurography for Evaluation of Peripheral Nerves

**DOI:** 10.1055/s-0041-1729176

**Published:** 2021-05-14

**Authors:** Vanessa Ku, Cameron Cox, Andrew Mikeska, Brendan MacKay

**Affiliations:** 1 Department of Orthopaedic Surgery, Texas Tech University Health Sciences Center, Lubbock, Texas, United States; 2 Department of Orthopaedic Surgery, University Medical Center, Lubbock, Texas, United States

**Keywords:** magnetic resonance neurography, MRN, MRI, peripheral nerve imaging, nerve evaluation, peripheral nerve injury

## Abstract

Peripheral nerve injuries (PNIs) continue to present both diagnostic and treatment challenges. While nerve transections are typically a straightforward diagnosis, other types of PNIs, such as chronic or traumatic nerve compression, may be more difficult to evaluate due to their varied presentation and limitations of current diagnostic tools. As a result, diagnosis may be delayed, and these patients may go on to develop progressive symptoms, impeding normal activity. In the past, PNIs were diagnosed by history and clinical examination alone or techniques that raised concerns regarding accuracy, invasiveness, or operator dependency. Magnetic resonance neurography (MRN) has been increasingly utilized in clinical settings due to its ability to visualize complex nerve structures along their entire pathway and distinguish nerves from surrounding vasculature and tissue in a noninvasive manner. In this review, we discuss the clinical applications of MRN in the diagnosis, as well as pre- and postsurgical assessments of patients with peripheral neuropathies.

## Introduction

Peripheral nerve injuries (PNIs) are common, with an incidence of 16.9 per 100,000 citizens in the United States. 1 Etiologies include traumatic injury, metabolic disorders, and chronic compression. 1 PNIs, particularly those that are not caused by trauma, are difficult to diagnose given that their presentation varies, and initial symptoms may be vague or misleading. Though impaired, many nerves retain some function and require a nuanced diagnostic approach. Early diagnosis is critical in these cases, as symptoms can rapidly progress from mild to debilitating if treatment is delayed and/or inappropriate for the particular injury.

Historically, suspected peripheral neuropathies have been diagnosed by clinical presentation, nerve biopsy, and nerve conduction studies (NCSs). 2 3 However, nerve biopsy is an invasive procedure, and NCSs are not helpful in the case of severe nerve degeneration. 2 When the cause of nerve dysfunction is known and patients fail nonoperative treatment, neurolysis, decompression, and/or nerve transpositions are often performed to treat symptoms and complications of PNIs. Even when the cause of dysfunction is understood, nerve assessments may still be necessary to identify the site of neurologic compromise for surgical planning. Recently, techniques have been developed to visualize peripheral nerves and supplement traditional diagnostic tools in complicated cases.

Visualization of nerve structures can help determine the location and/or cause of nerve dysfunction. 2 Furthermore, quantification of axonal and myelin degeneration and regeneration allows monitoring of disease progression both pre- and posttreatments. 2 While ultrasound has been used to visualize superficial nerves, quality of these images is dependent on technician skill. 2

In 1992, Filler et al introduced magnetic resonance neurography (MRN), an imaging technique that takes advantage of nerve tissue's longer T2 isolation time. 4 Through homogenous fat suppression, blood signal intensity suppression, and heavy T2 weighting, the nerve can be shown in relative isolation, brighter than its surrounding tissue. 5 6

MRN is becoming more frequently employed as it is noninvasive and allows physicians to differentiate between neurologic and nonneurologic disorders and locate damaged nerve segments to narrow the differential diagnosis. 3 The use of serial MRN may also allow physicians to track the progression of symptoms and recovery. In this review, we discuss the clinical applications of MRN in diagnosis, treatment, and posttreatment monitoring of both chronic and traumatic peripheral neuropathies.

## Methods

The authors performed a systematic review of the MEDLINE and EMBASE databases using a comprehensive combination of keywords and search algorithm according to the Preferred Reporting Items for Systematic Reviews and Meta-Analyses guidelines. The literature search focused on clinical data regarding MRI and MRN imaging modalities for peripheral nerves and was undertaken to define the current understanding of MRN as a method of evaluating nerve structure and function.

## Brachial Plexus

Due to the depth of the brachial plexus and its complex anatomy, brachial plexus lesions are often difficult to characterize and treat. 7 Furthermore, conditions such as cervical spondylosis-related radiculopathy or cervical disc herniation present similar to brachial plexopathies. 7 8 9 Thus, a precise technique is required for evaluation. MRN has become a valuable diagnostic tool for brachial plexopathies caused by trauma, acute or chronic inflammation, brachial plexus tumors, and thoracic outlet syndrome 10 11 as it can locate the site of neurologic compromise with a high degree of precision.

A common context for diagnostic use of MRN is Parsonage–Turner's syndrome (PTS), also known as acute idiopathic brachial plexus neuritis. PTS is broadly characterized by the inflammation of nerves in the chest, shoulders, and arms which commonly presents as pain and flaccid paralysis of the corresponding musculature. 8 12 One case report described a 55-year-old woman with right-sided, burning, scapular pain which radiated down her right arm with numbness to the first three fingers of her right hand. MRN of the right brachial plexus revealed a mild increase in intensity and thickening of the C7 root, middle trunk, and posterior cord, which lead to the diagnosis of PTS. 12 A study of 15 PTS patients presenting with a history of weakened shoulder abduction, MRN analysis revealed the involvement of roots in 8 (53.5%) patients, trunks in 7 (46.7%) patients, cords in 6 (40%) patients, and terminal branches in 2 (13.3%) patients. 8 MRN was also used to assess the condition of the muscles innervated by the brachial plexus. Edema, fatty infiltration, and atrophy were detected in eight (53.3%) patients. In both reports, MRN was a useful tool in the visualization of the entire brachial plexus pathway, from the roots to the terminal branches. 8

In addition to its diagnostic utility, MRN has shown promise in evaluating postsurgical recovery via T2 nerve signal intensity and signs of muscle degeneration. One article described three patients with C5 to C7 injuries who underwent Oberlin transfer (partial ulnar nerve to biceps transfer to restore elbow flexion). 13 14 Postoperative MRN, which showed normal findings in the biceps muscle, was performed to visually assess the results of the surgery by looking at the T2 signal intensity of nerves and signs of muscle degeneration. 14 Functional recovery of the biceps muscle was confirmed through absence of neurogenic muscle edema or biceps atrophy on MRN at final follow-up. 14

## Upper Extremity Nerve Entrapments

Upper extremity (UE) nerve entrapments often present with mild symptoms, such as numbness or tingling that can progress to pain and/or functional deficits. 15 In some cases, symptoms may deviate from the classic presentation for compressive neuropathy. Unfortunately, diagnosis may be delayed or missed altogether due to the difficulty of discerning the underlying cause of clinical symptoms. Ulnar and median nerve entrapment can progress to atrophy and loss of function in the intrinsic hand muscles in advanced stages, and early diagnosis is needed to maximize functional recovery. Currently, there are limited data assessing MRN as a means of evaluating UE nerve entrapments with literature ranging from case reports to prospective studies of MRN in median and ulnar nerve compression.

On MRN, a normal median nerve appears hypointense on a T1-weighted (T1W) image and isointense to minimally hyperintense on a T2-weighted (T2W) image along the course of the arm. 16 Deviations from the norm may be interpreted as signs of an underlying condition. 17 18 19 For example, findings of chronic nerve entrapment on MRN include nerve swelling proximally and sometimes distally with an abrupt transition to a flattened contour at the entrapment site. 20

Aggarwal et al described a case of an 18-year-old man presenting with a medial epicondyle fracture and elbow dislocation treated via closed reduction of the elbow and cast immobilization. After cast removal, he reported progressive numbness and paresthesia over the median nerve distribution of the forearm and hand with inability to form a fist. NCS revealed median nerve dysfunction at the elbow, but did not identify the exact site of entrapment. 15 MRN showed median nerve entrapment between the olecranon of the ulna and olecranon fossa of the humerus. The muscles of the forearm in the anterior compartment displayed hyperintense signals on T1W and T2W sequences, suggesting chronic denervation with fatty infiltration. 15 Results of MRN lead to the diagnosis of median nerve entrapment within the medial epicondyle (Type 2). 15 Given that potential treatments for median nerve entrapment differ in early versus late detection, MRN may be a useful tool in the diagnostic algorithm. 15 In this particular injury pattern, early detection allows relocation of the nerve from posterior to anterior compartment. However, delayed detection results in excision of the affected segment with end-to-end repair or grafting, both of which have a poorer prognosis than early relocation. 15

Carpal tunnel syndrome (CTS) is a common cause of median nerve entrapment. Bao et al conducted a study of 47 CTS patients who were diagnosed primarily by clinical examination with a positive Tinel's and Phalen's signs, followed by NCS and electromyography (EMG). 21 While these diagnostic tools can raise suspicion for CTS, diffusion-weighted MRN (DW-MRN) can directly visualize and locate median nerve lesions in wrists of CTS patients. 21 In this cohort, DW-MRN revealed obvious median nerve hyperintensity, median nerve compression, and enhanced flexor retinaculum bowing. 21 The DW-MRN mean apparent diffusion coefficient (ADC) of median nerves in the CTS group (1.13 × 10 ^−3^ mm ^2^ /s) was significantly higher than that of the control group (1.06 × 10 ^−3^ mm ^2^ /s) ( *p* < 0.01). 21 These ADC values might be used to establish ADC cutoffs and/or contralateral comparison studies for improved sensitivity of CTS diagnosis.

In a study of 30 CTS patients, MRN images taken prior to decompression surgery showed an increased signal change in the proximal median nerves, proximal nerve swelling at the level of the pisiform and distal radius, and increased flattening of the distal median nerve in the majority of CTS patients, compared with the healthy controls. 22 MRN images taken 3 months after surgery showed a return to normal nerve signal and a reduction in size of the nerve toward control values in the majority of CTS patients, compared with the healthy controls. 22 Furthermore, in the distal part of the carpal tunnel where the median nerve was most compressed, there was a significant increase in the cross-sectional area of the median nerve in all patients studied. 22 While electrophysiological readings prior to surgical decompression were strongly suggestive of CTS, these readings remained abnormal 3 months postsurgery, whereas MRN confirmed successful median nerve decompression. 22

Similar to the median nerve, ulnar nerves show changes in both T1W and T2W intensities when damaged, particularly in the wrist region. 23 24 Variations in T1W and T2W signals on MRN can help delineate between different causes of ulnar neuropathy, including trauma, overuse (chronic compression), arthritis, masses and mass-like lesions, and systemic diseases. 23 MRN has been used to identify conditions not previously suspected. 23 24 25

Chhabra et al reported a case of a 46-year-old woman with a history of five ulnar nerve surgeries on the right elbow that presented with many months of pain and paresthesia over the ulnar nerve distribution in her forearm and hand as well as decreased pinch and grip strength, which began after a submuscular ulnar nerve transposition—her most recent surgery. 25 MRN showed an enlarged ulnar nerve with mild perineural fibrosis and mild hyperintensity as it exited the medial intermuscular septum, suggesting an ulnar nerve re-entrapment. 25 Additionally, the median nerve displayed mild hyperintensity and minimal perineural fibrosis, and the medial antebrachial cutaneous (MABC) nerve was focally enlarged in the distal arm, indicating a neuroma. 25 These findings were confirmed intraoperatively. While EMG showed moderate ulnar neuropathy, it did not indicate median nerve abnormality. 25 In this case, MRN helped identify two conditions not suspected preoperatively—MABC neuroma, which can mimic ulnar neuropathy, and median nerve entrapment, likely due to previous surgery-related fibrosis. 25

Recently, diffusion tensor imaging (DTI) and tractography have been used in conjunction with MRN to detect proximal lesions and distinguish between multifocal and focal-compressive ulnar neuropathy at the elbow in cases where clinical examination and EMG/NCS were unable to localize the site of nervous deficiency. 26 27 In a study of 122 patients presenting with ulnar mononeuropathy of unknown etiology and localization (after clinical examination and electrophysiological testing), MRN with T2W fat-saturated sequences revealed proximal lesion extension with significantly increased T2W signal in the upper arm in 21 patients. 26 In the group with an additional proximal lesion, 10 had undergone previous decompression surgery for ulnar nerve entrapment at the elbow. Of note, 10 patients also had previously undetected lesions in the median and/or radial nerve(s) which were more pronounced in the upper arm but still visible at the elbow on MRN. 26

Current literature suggests that MRN is a more precise tool than NCS and EMG in determining the exact site of UE nerve entrapment and assessing nerve recovery following surgical intervention. Furthermore, when symptoms persist despite attempted interventions, MRN can be used to augment the diagnostic algorithm and may reveal pathologies not previously suspected based on physical examination and NCS/EMG.

## Lower Extremity Neuropathy

Patients with lower extremity entrapment neuropathy, an under-recognized cause of pain and functional impairment, often present with nonspecific symptoms, which makes it difficult to distinguish from more common, nonneurologic causes of pain. 28 MRN has been used to evaluate causes of tibial nerve dysfunction, such as tarsal tunnel syndrome, Morton's neuroma, median plantar nerve entrapment, and lateral plantar nerve compression. 29 It has also been used to visualize femoral nerve abnormalities caused by conditions including lumbar plexopathy, nerve sheath tumors, trauma, and chronic inflammatory demyelinating polyradiculoneuropathy. 30

While various lower extremity nerves have been described in the literature, the majority of reports address causes of sciatic neuropathies. Intra- or extra-articular hip pathology and lumbar radiculopathy present with symptoms similar to sciatic nerve entrapment. 31 As a result, posterior hip pain and sciatica frequently present diagnostic and therapeutic challenges, and a precise diagnostic tool is needed to focus the differentials. 32 Sciatic neuropathy has traditionally been diagnosed using a combination of clinical history, physical examination, and electrodiagnostic studies. 33 However, MRN is becoming increasingly popular in the assessment of sciatic neuropathy due to its multiple advantages, including assessment of nerve continuity, localization of injury or entrapment, and detection of secondary muscle denervation. 33 34 MRN helps determine the cause of sciatic neuropathy by identifying piriformis muscle asymmetry and sciatic neuromuscular variants through visualization of the sciatic nerve as it traverses the posterior leg. 35 The literature regarding MRN's utility in sciatic nerve evaluation ranges from case reports describing unique presentations to prospective studies investigating the ability of MRN to address shortcomings of traditional diagnostic tools. 36

Chitranjan et al described the case of a 55-year-old woman with 2 years of left gluteal swelling and left lower extremity pain. A computed tomography scan revealed herniation of the sigmoid colon through the greater sciatic foramen and atrophy of the gluteal muscles. 36 To confirm a suspected entrapment of the sciatic nerve, MRN was performed, which revealed thickening and increased signal in the left sciatic nerve, as well as lateral deviation and entrapment of the nerve by the hernia and atrophy of the left gluteal muscles. 36 In this case, MRN was successfully employed to demonstrate the pathway and entrapment of the sciatic nerve.

Polesello et al reported a case of a 42-year-old woman with 17 years of recurrent left-sided low back pain, which was initially diagnosed as L5-S1 spondylolisthesis with a herniated disc. Despite treatment with physiotherapy, analgesics, and an L5-S1 arthrodesis, the patient's pain became progressively worse, preventing her from work. 37 Five subsequent MRI examinations revealed no abnormal findings in the lumbosacral spine and left hip. 37 MRN over the left hip region eventually revealed an accessory muscle belly of the left piriformis with the fibular branch of the sciatic nerve passing between the fibers of this accessory belly and the standard piriformis muscle. 37 In this case, MRN provided a definitive diagnosis regarding the etiology of the patient's pain, which subsequently allowed accurate presurgical planning for endoscopic release of the piriformis. 37 38 39 40

One prospective study included 239 patients presenting with leg pain in the sciatic nerve distribution and either inconclusive diagnosis or unsuccessful prior lumbar spine surgery. 38 MRN was performed on all patients and was able to distinguish patients with piriformis syndrome (from patients with similar symptoms) with 93% specificity and 64% sensitivity. 38

Recent studies have employed the use of MRN in diagnosing lower extremity nerve abnormalities that may not have been detected by alternative methods such as MRI, NCS, and EMG. In the case of sciatic neuropathy, MRN can be used to determine the site neurologic compromise and distinguish between its various causes such as piriformis syndrome or compression due to trauma.

## Diabetic Neuropathy

Diabetic peripheral neuropathy (DPN) is common in diabetics and can cause irreversible nerve damage. DPN also increases risk of diabetic foot ulcers which, if left untreated, may lead to amputation. 41 42 Historically, changes in the nerve microstructure in the lower extremity peripheral nervous system have been evaluated by NCS and nerve biopsy, both of which measure structural changes indirectly. 43 To prevent progression of DPN, noninvasive diagnostics with high sensitivity are needed for early detection.

MRN studies of patients with type 1 diabetes (T1D) and type 2 diabetes (T2D) have shown that T1D patients with DPN have a higher T2W hyperintense lesion load compared with T2D patients with DPN, and T2D patients with DPN have a higher T2W hypointense lesion load compared with T1D patients with DPN. 44 45 This was found to be a positive correlation between the amount of both lesion types and the severity of clinical symptoms, as DPN patients with intraneural T2 lesions had higher neuropathy deficit scores than DPN patients without intraneural T2 lesions. 45 46 T2W hyperintense lesions have also demonstrated a positive correlation with the impairment of nerve conduction parameters and HbA1c levels. 45 Thus, MRN can be utilized to distinguish between T1D and T2D, by taking advantage of their different mechanisms of nerve damage.

Furthermore, studies have shown that lesions extend over longer distances in patients with painful DPN versus those with nonpainful DPN or no DPN, and the maximum length of a lesion is positively correlated with the per cent coverage of T2W hyperintense nerve lesions along a full nerve. 44 Fractional anisotropy (FA) values decrease and ADC values increase with the severity of neuropathy. 42 47 Multiple MRN studies have shown that despite DPN symptoms presenting with a strong distal focus, there is a proximal nerve lesion predominance at the thigh level as well as a higher discriminatory power in FA and ADC values at the sciatic level. 42 43 Diabetics without DPN have similar FA and ADC values as healthy controls, suggesting that MRN can be used to distinguish between diabetics with and without DPN. 47

MRN is a promising imaging tool that may be employed as a diagnostic marker in the early detection of DPN in diabetic patients. Early detection may assist in preventing the progression of DPN to debilitating conditions that negatively impact quality of life.

## Traumatic Nerve Injury

PNI, including brachial and lumbar plexus injuries, occurs in ∼5% of all traumas. 48 These injuries are most often a result of motor vehicle accidents, falls, or penetrating trauma. 49 The radial, ulnar, and median nerves are the most commonly injured UE nerves, and the sciatic, peroneal, femoral, and tibial nerves are most often affected in lower extremities. 50 Delayed diagnosis of PNI may be attributed to a variety of comorbid factors. An ischemic or badly injured limb can complicate physical examination. 48 In bedridden patients, lower extremity nerve defects may not clearly manifest until the patient is mobilized. 48 These PNIs can have a delayed presentation, secondary to musculoskeletal injuries, such as compression from hematoma, compartment syndrome, or complications from management of concurrently injured tissues. 48 Failure to address injured nerves in a timely manner can lead to long-term functional deficits. When physical examinations are inconclusive or highlight a high index of suspicion, early detection using MRN can prevent permanent damage that could lead to decreased strength, limited range of motion, or loss of sensation and/or proprioception. 51 52

After closed injuries, it may be difficult to distinguish between nerve injuries that have the potential to recover on their own (neurapraxic and axonometric) from those that do not (neurotmetic) and require surgery. 53 While both axonometric and neurotmetic nerve injuries are characterized by absent nerve conduction responses and muscle denervation on both EMG and MRI, neuropraxic injuries exhibit no EMG or MRI signs of muscle denervation. 53 Using EMG and NCS, it can take 2 to 3 weeks to detect signs of muscle denervation, whereas MRN can detect muscle signal alterations as early as 4 days postinjury, indicating that MRN may be better suited to early detection of muscle denervation. 52 53

One study of 20 patients with features of traumatic brachial plexopathies assessed correlation between MRN and subsequent intraoperative findings. 54 MRN findings included edema, scarring, pseudo-meningoceles, and neuromas, which were detected at the levels of the roots, trunks, and cords. 54 These findings were given a score based on whether they correlated with operative findings at all three levels, any two levels, or any one level. 54 When comparing MRN readings to intraoperative findings, 13 patients received a score of 3 (good correlation), 6 patients received a score of 2 (average correlation), and 1 patient received a score of 1 (poor correlation), supporting the use of MRN for discerning the location and extent of traumatic brachial plexus injuries. 54

In another study, 11 infants with birth-related brachial plexus injuries were assessed to compare MRN versus EMG, since EMG is a painful procedure done without sedation and can be difficult to perform in children. MRN findings included muscle denervation changes, T2 prolongation along the affected brachial plexus, and enhancement or thickening of nerve roots. 55 MRN revealed nerve injuries that ranged from neuropraxia to avulsion, a preganglionic tear that requires microsurgical repair before 3 months of age for the best chance of recovery. 55 Furthermore, there was a greater degree of correlation between MRN and physical examination findings than between EMG and physical examination findings, supporting MRN's efficacy in differentiating the type of nerve injuries for presurgical planning. 55

Advantages of MRN include early characterization of the location and grade of nerve injury and improved accuracy when compared with NCS and EMG. Published data suggest that MRN could be a valuable addition to the diagnostic algorithm for traumatic nerve injuries, especially when traditional assessment tools are not able to accurately diagnose nerve conditions.

## Discussion

Delayed diagnosis of PNI leads to delayed treatment, thus narrowing the scope of available treatment options. Ultimately, this can result in long-lasting functional deficits and impaired quality of life. 51 56 Historically, PNIs have been diagnosed by clinical symptoms and indirect and/or invasive methods of evaluating nerve function, such as NCS and EMG. 51 56 While these tools remain essential to diagnostic and postoperative workups, they do not adequately address the full spectrum of nerve injuries seen in a clinical setting. Given the gaps in the current nerve assessment algorithm, MRN has recently been used to supplement traditional techniques for both pre- and postoperative characterization of nerve structures. MRN can provide high-quality images of structures in difficult anatomical areas without a skilled operator, locate the precise location of nerve injury, and visualize signs of secondary muscle denervation, thus addressing many shortcomings of the current diagnostic/monitoring algorithm. 20 28 51 Injured nerves will exhibit hyperintense signal on T2W images within 24 hours of injury, reflecting a nonspecific response of the nerve to injury. 17 28 In the case of nerve entrapment, nerve hyperintensity is most prominent at the site of entrapment, allowing localization of the nerve lesion. 17

MRN also provides excellent soft tissue contrast, allowing for visualization of downstream muscle injury and high contrast resolution between surrounding fat and vascular structures. 20 Signs of muscle denervation can be visualized distal to the site of nerve injury with diffuse muscle signal alterations and without hemorrhage or fascial edema. 17 In an acute setting, often within 48 hours of injury, denervated muscle displays hyperintense signals on T2W images due to increased extracellular fluid and edema. 20 57 In chronic muscle denervation, due to volume loss and fatty infiltration, denervated muscle displays hyperintense signals on T1W images, indicating irreversible end-stage disease and muscle atrophy. 20 28 Thus, being able to understand the extent and duration of a nerve deficit through the differentiation of acute versus chronic muscle denervation on MRN allows physicians to make better informed decisions about the time frame of surgical intervention.

Recently, there has been an increased interest in combining MRN with DTI and diffusion-weighted imaging (DWI). DWI can be used to study the extracellular movement of water, and it capitalizes on the difference in diffusion properties in various types of tissue. 20 51 Thus, DWI can allow qualitative assessment of axonal fiber integrity. 58 DTI, unlike DWI, provides more quantitative data on water diffusion and axonal conduction along a nerve via the acquisition of multiple diffusion directions. 58 Both modalities can enhance the quality of peripheral nerve imaging by showing barriers, such as the myelin sheath, that allow water movement along the longitudinal axis rather than the perpendicular axis. 20 51

Nerve-specific contrast agents such as Gadofluorine M, though not yet used clinically, have shown promise in nerve imaging. 20 59 These contrast agents contain iron oxide, which creates a hypointense signal in T2W sequences which is localized to sites of nerve inflammation. 20 In animal studies, Gadofluorine M has been shown to accumulate in nerve fibers undergoing Wallerian degeneration and disappear upon remyelination. 59 Nerve contrast agents such as Gadoflourine M are a promising next step in the direct visualization of nerve structures for preoperative diagnosis and evaluation of postsurgical outcomes.

## Conclusion

MRN has been utilized in a variety of nerve injury patterns to provide insights beyond traditional assessment modalities. The current literature indicates that MRN has the potential to improve clinical outcomes, particularly in nerve defects that are either difficult to detect or require early intervention.
